# Supplementary material for: Movement ecology of captive-bred axolotls in restored and artificial wetlands: Conservation insights for amphibian reintroductions and translocations
Source: PLoS One. 2025 Apr 30;20(4):e0314257. doi: 10.1371/journal.pone.0314257 (PMC12043180; doi:10.1371/journal.pone.0314257)
Supplement: S1 Table — The table includes the mean, standard deviation (Std Dev), minimum (Min), and maximum (Max) values for pH, dissolved oxygen (DO, expressed as both percentage saturation and concentration in mg/L), conductivity (µS/cm), and salinity. Data were collected daily on monitoring days throughout the duration of the study. (DOCX) [file pone.0314257.s001.docx]

|  | **LCO** | | | | **Xochimilco** | | | |
| --- | --- | --- | --- | --- | --- | --- | --- | --- |
| **Parameter** | **Mean** | **Std Dev** | **Min** | **Max** | **Mean** | **Std Dev** | **Min** | **Max** |
| **pH** | 9.23 | 0.94 | 7.07 | 10.58 | 9.11 | 0.61 | 7.01 | 9.86 |
| **Dissolved Oxygen (OD%)** | 39.73 | 22.28 | 5.7 | 92.6 | 22.27 | 25.3 | 0.04 | 112.2 |
| **Dissolved Oxygen (OD mg/L)** | 4.12 | 2.27 | 0.62 | 9.39 | 1.62 | 1.9 | 0.02 | 9.74 |
| **Conductivity (µS/cm)** | 324.94 | 3.62 | 319 | 336 | 1266.83 | 244.66 | 892 | 1797 |
| **Salinity (sal)** | 0.15 | 0.004 | 0.15 | 0.16 | 0.64 | 0.13 | 0.44 | 0.92 |
