# Supplementary material for: Movement ecology of captive-bred axolotls in restored and artificial wetlands: Conservation insights for amphibian reintroductions and translocations
Source: PLoS One. 2025 Apr 30;20(4):e0314257. doi: 10.1371/journal.pone.0314257 (PMC12043180; doi:10.1371/journal.pone.0314257)
Supplement: S2 Table — Summary of individual characteristics of axolotls studied across two different study areas, LCO and Xochimilco. The table lists the identification code (ID), sex, age (in years), body mass (in grams), and length (in centimeters) of each axolotl. (DOCX) [file pone.0314257.s002.docx]

| **Study Area** | **ID** | **Sex** | **Age** | **Mass** | **Length** |
| --- | --- | --- | --- | --- | --- |
| LCO | A01 | female | 2.45 | 63.9 | 21 |
| LCO | B12 | female | 5.61 | 59.4 | 23 |
| LCO | C10 | female | 2.19 | 73.9 | 21 |
| LCO | A09 | female | 2.45 | 72.6 | 21 |
| LCO | A12 | male | 2.36 | 67.2 | 22 |
| LCO | C07 | male | 4.11 | 78.9 | 24 |
| LCO | C12 | male | 2.45 | 68.4 | 21 |
| LCO | A16 | male | 2.53 | 80.7 | 24 |
| Xochimilco | A08 | female | 2.56 | 85.3 | 23 |
| Xochimilco | A15 | female | 3.98 | 97.6 | 24 |
| Xochimilco | B02 | female | 1.39 | 68.2 | 21 |
| Xochimilco | B06 | female | 1.56 | 97.4 | 23 |
| Xochimilco | B14 | female | 2.56 | 75.2 | 21 |
| Xochimilco | A10 | male | 1.39 | 59.8 | 21 |
| Xochimilco | C03 | male | 4.39 | 86.8 | 28 |
| Xochimilco | C05 | male | 2.56 | 64.3 | 23 |
| Xochimilco | C09 | male | 2.73 | 82.7 | 24 |
| Xochimilco | C11 | male | 1.56 | 78.6 | 23 |
