# Supplementary material for: Movement ecology of captive-bred axolotls in restored and artificial wetlands: Conservation insights for amphibian reintroductions and translocations
Source: PLoS One. 2025 Apr 30;20(4):e0314257. doi: 10.1371/journal.pone.0314257 (PMC12043180; doi:10.1371/journal.pone.0314257)
Supplement: S3 Table — Comparison of individual traits (mass, length, and age) and home range (MCP and KDE 50%) between axolotls from LCO and Xochimilco, and between sexes within each location. Values are presented as means with ranges in parentheses. Significant p-values are highlighted in bold. (DOCX) [file pone.0314257.s003.docx]

| **Parameters** | **Mass (grams)** | **Length (cm)** | **Age (years)** | **MCP**  **(m²)** | **KDE 50% (m²)** |
| --- | --- | --- | --- | --- | --- |
| LCO |  |  |  |  |  |
| average | 70.63 | 22.09 | 3.02 | 2747 | 1640 |
| range | (59.4-80.7) | (20.6-24) | (2.2-5.6) | (548-4402) | (211-3673) |
| Xochimilco |  |  |  |  |  |
| average | 79.59 | 23 | 2.47 | 382 | 204 |
| range | (59.8-97.6) | (20.9-27.5) | (1.4-4.4) | (175-697) | (35-455) |
| LCO vs Xochimilco |  |  |  |  |  |
| p-value | 0.1 | 0.45 | 0.82 | **0.00018** | **0.0014** |
| Females LCO |  |  |  |  |  |
| average | 67.44 | 21.43 | 3.18 | 3096 | 1933 |
| range | (59.4-73.9) | (20.6-22.9) | (2.2-5.6) | (1705-4227) | (962-2771) |
| Males LCO |  |  |  |  |  |
| average | 73.82 | 22.75 | 2.86 | 2397 | 1347 |
| range | (67.2-80.7) | (21.2-24) | (2.4-4.1) | (548-4404) | (211-3673) |
| Females Xochimilco |  |  |  |  |  |
| average | 87.13 | 22.7 | 2.37 | 443 | 220 |
| range | (68.2-97.6) | (20.9-23.5) | (1.4-3.9) | (179-697) | (36-455) |
| Males Xochimilco |  |  |  |  |  |
| average | 73.4 | 23.83 | 2.77 | 319 | 188 |
| range | (59.8-86.8) | (21-27.5) | (1.4-4.4) | (174-567) | (66-256) |
| Females vs Males |  |  |  |  |  |
| LCO p-value | 0.24 | 0.24 | 0.88 | 0.68 | 0.49 |
| Xochimilco p-value | 0.23 | 0.46 | 0.83 | 0.31 | 0.99 |
