# Supplementary figures and images for: Movement ecology of captive-bred axolotls in restored and artificial wetlands: Conservation insights for amphibian reintroductions and translocations
Source: PLoS One. 2025 Apr 30;20(4):e0314257. doi: 10.1371/journal.pone.0314257 (PMC12043180; doi:10.1371/journal.pone.0314257)

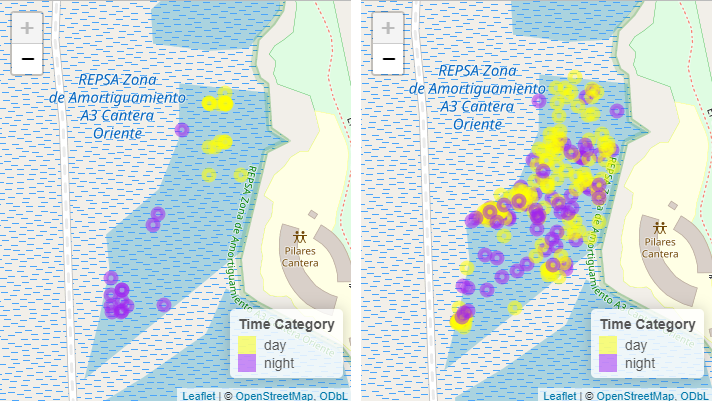

Supplement: S4 Fig — This figure displays the spatial distribution of axolotl observation points in La Cantera Oriente (LCO). The pilot study data are shown on the left, and the current study data on the right. Yellow points represent axolotl locations observed during the day, while purple points denote nighttime locations. This map was created using was created using OpenStreetMap data, which is licensed under the Open Database License (ODbL). (TIFF) [file pone.0314257.s004.tiff]
