# Supplementary material for: Movement ecology of captive-bred axolotls in restored and artificial wetlands: Conservation insights for amphibian reintroductions and translocations
Source: PLoS One. 2025 Apr 30;20(4):e0314257. doi: 10.1371/journal.pone.0314257 (PMC12043180; doi:10.1371/journal.pone.0314257)
Supplement: Attachment — Submitted filename: Response to Reviewers.docx [file pone.0314257.s007.docx]

**Response to Reviewers and Editors**

We sincerely appreciate the thoughtful and constructive feedback from the reviewers and the editor. Their insights have helped us improve the clarity and quality of our manuscript. Below, we provide our detailed responses to each comment and indicate the corresponding revisions made to the manuscript.

**Line 27.-** I do not believe that the concept of the Mexican axolotl is well applied since there are more species of axolotls in Mexican territory, so the best concept is the Xochimilco axolotl.

We appreciate the reviewer’s observation. However, referring to *Ambystoma mexicanum* as the "Xochimilco axolotl" could be misleading, as this is not a widely recognized common name in the scientific literature.

The IUCN Red List refers to this species simply as "axolotl," while other reputable sources, including AmphibiaWeb, Amphibian Species of the World, and Britannica, commonly use either "axolotl" or "Mexican axolotl." Additionally, a Google Scholar search for *Ambystoma mexicanum* paired with 'Xochimilco axolotl' yields only 11 results, whereas pairing it with 'Mexican axolotl' returns 2,520 results, further indicating the limited use of 'Xochimilco axolotl' in scientific publications.

Using "Xochimilco axolotl" may also unintentionally suggest that it is a distinct species or subspecies, which is not the case. However, we acknowledge the reviewer’s important point that multiple *Ambystoma* species exist in Mexico. To address this, we have revised the manuscript to explicitly state that Mexico is home to 17 *Ambystoma* species while distinguishing *A. mexicanum* as the species most commonly referred to as "axolotl."

Additionally, we now explain that *Ambystoma dumerilii* and *Ambystoma andersoni*, two other Mexican *Ambystoma* species, are locally called "achoque," a term from the Purépecha language that reflects their cultural and regional significance in the Lake Pátzcuaro region.

Finally, for consistency and clarity, we have replaced "Mexican axolotl" with "axolotl" throughout the manuscript.

References:

- [IUCN Red List](https://www.iucnredlist.org/species/1095/53947343)
- [Amphibian Species of the World](https://amphibiansoftheworld.amnh.org/Amphibia/Caudata/Ambystomatidae/Ambystoma/Ambystoma-mexicanum?)
- [Britannica](https://www.britannica.com/animal/axolotl)
- [AmphibiaWeb](https://amphibiaweb.org/cgi/amphib_query?where-genus=Ambystoma&where-species=mexicanum&utm)

**Line 28.-** specify Xochimilco lake since putting only Xochimilco refers to the mayor's office or municipality.

Thank you for the suggestion. We replaced “Xochimilco” with “Lake Xochimilco”.

**Line 36.-** specify values of how much they traveled or on average with the males

Thank you for your suggestion. We have now included the average daily distances traveled by each sex in the revised text. Specifically, we state that males traveled an average of 54.33 meters per day, while females traveled 86.75 meters per day.

**Line 54.-** update quote speaks in the present tense and it's been 5 years, they suggest that half of these species are probably also threatened so I think the statistic has already changed.

We appreciate the suggestion to update this information, and in response, we have revised the sentence to clarify that this statistic was from 2019.

**Line 56-58.** Habitat loss, fragmentation and degradation are among the main drivers of extinctions and declines in amphibian populations worldwide, but pollution of ecosystems is missing.

We appreciate the reviewer’s observation. Since pollution is generally considered a form of habitat degradation, it was originally encompassed within that term. However, to ensure clarity, we have now revised the sentence to explicitly acknowledge pollution as a contributing factor.

**Line 62.** Refer to Ambystoma mexicanum as the Xochimilco axolotl or Axolotl, since there are more species of axolotls in Mexico

We appreciate the reviewer’s suggestion. As noted in our previous response, we have opted to use "axolotl" throughout the manuscript for consistency with widely accepted terminology in the scientific literature.

**Line 70-73.** Emphasize current problems, it is read that this happened in pre-Hispanic times

We appreciate the reviewer’s suggestion. To clarify that these are ongoing issues rather than historical ones, we have revised the text to explicitly state that Xochimilco currently faces severe environmental challenges, including urban expansion, environmental degradation, invasive species, and declining water quality (Lines 79 – 82).

**Line 73-74.** Who is this project from, who participates, what institutions?

We appreciate the reviewer’s request for clarification. We have revised the text to specify that the *chinampa-refuge* project was initiated in 2004 by the Laboratorio de Restauración Ecológica at the Universidad Nacional Autónoma de México (UNAM). Additionally, we clarified that the project is carried out in collaboration with local *chinampero* farmers.

**Line 76.** Why is the water quality so poor?

We appreciate the reviewer’s request for more details. To clarify, we have revised the text to specify that water quality deterioration in Xochimilco is primarily caused by untreated wastewater discharge, agricultural runoff, and reduced water exchange due to urban infrastructure modifications.

**Line 80.** Emphasize that translocation is one of those continuous and proactive human interventions to preserve vulnerable species and prevent extinctions

We appreciate the reviewer’s suggestion. We have revised the text to explicitly emphasize translocation as a continuous and proactive intervention in amphibian conservation (Lines 90 – 92).

**Line 91-92.** Explain the importance of what you mention because the habitat of Ambystoma mexicanum is wetlands (Lake Xochimilco)

Thank you for pointing this out. We have revised the text to better emphasize the connection between wetland restoration efforts and the conservation of the Mexican axolotl (*Ambystoma mexicanum*), whose natural habitat includes wetlands like Lake Xochimilco (Lines 108 – 109).

**Line 105-106.** We all know that the conservation of endangered species within their natural habitats remains the ideal strategy, but why?

Thank you for this comment. We have revised the text to briefly explain why conserving species within their natural habitats is considered the ideal strategy (Lines 125 – 127).

**Line 106.** Mention Lake Xochimilco, not just Xochimilco because you are referring to the aquatic system, not the mayor's office, please check this throughout the document

Thank you for your suggestion. We now refer to *Lake Xochimilco* throughout the Introduction to clarify that we are discussing the aquatic system. However, in other sections of the manuscript, we refer to our specific study area—a restored chinampa within Lake Xochimilco—as *Xochimilco* to maintain accuracy and avoid potential confusion about the spatial scale of our study. To ensure clarity for readers, we have now explicitly defined this in the Methods section:

*"This study was conducted in two distinct aquatic habitats located in southern Mexico City: a restored chinampa in Lake Xochimilco (hereafter Xochimilco) and a pond in the artificial wetland of La Cantera Oriente (LCO) (Fig 1).*

This definition allows us to use *Xochimilco* consistently when referring to the study site while ensuring that readers do not confuse it with the entire lake system. We are more concerned that referring to *Lake Xochimilco* throughout the manuscript could lead readers to mistakenly assume that our study covered a larger portion of the lake rather than our specific study site.

**Line 109.** Justify why you mention the eastern quarry, you only mention it suddenly, can you mention, LIKE THE CASE OF……, AN AREA………

Thank you for this observation. We have revised the text to provide more context and justify the inclusion of La Cantera Oriente (LCO) by highlighting its relevance as an example of how artificial wetlands can support biodiversity and contribute to conservation efforts.

**Line 117-119.** You mention that given these characteristics, LCO could function as a potential habitat for axolotls, but you mention that it has rivers, lakes, fauna and flora, but what makes it ideal, for example temperature, water pH, is it not waste water, does it have invasive species?, the conductivity is perfect, the oxygen levels are similar to the demand of the organisms etc etc, why is it an alternative site for conservation efforts outside of its native environment?

We appreciate the reviewer’s insightful suggestion regarding the need to clarify the suitability of La Cantera Oriente (LCO) as a potential habitat for axolotls. In the revised manuscript, we have added detailed information to explain why LCO could be an alternative site for conservation efforts outside of the axolotl’s native habitat. Specifically, we now highlight physicochemical parameters, food availability, and evidence of axolotl reproduction and survival (Lines 135 – 140).

**Line 121- 131.** I consider the physicochemical factors, which I do not see described in this paragraph or analyzed for a study of this type, as they are crucial.

We appreciate the reviewer’s observation regarding the importance of physicochemical factors, and we agree that they are indeed crucial for understanding habitat suitability for axolotls. To address this, we would like to clarify that we conducted a prior study specifically focused on monitoring physicochemical parameters across all ponds in La Cantera Oriente (LCO). This allowed us to identify and select the pond with the most favorable conditions for axolotl reproduction and survival.

In the present study, we continued to monitor physicochemical parameters, including temperature, pH, and dissolved oxygen, throughout the monitoring periods. These data are summarized in the supplementary materials (S1 Table). Additionally, temperature was included as an explanatory variable in our analyses, as detailed in the methods section, and was a significant factor influencing axolotl movement as detailed in the results and discussion sections.

We have revised the manuscript (Lines 138–139) to explicitly state the specific factors registered in our study.

Line 136. Place Lake Xochimilco
Line 144. Place Lake Xochimilco

Line 151. In Lake Xochimilco we use ……

Line 237. Place Lake Xochimilco

Line 332. Indicate Lake Xochimilco

Thank you for these suggestions. In line **158**, we explicitly define our study site as a restored chinampa in Lake Xochimilco (hereafter Xochimilco) to ensure clarity. Since this definition is provided at the start of the Methods section, we refer to the study site as Xochimilco throughout the manuscript to maintain consistency and avoid unnecessary repetition of Lake Xochimilco.

As our study does not encompass the entire lake but rather a specific restored chinampa within it, we believe that continuously using *Lake Xochimilco* could mislead readers into thinking our study covered a broader area than it actually did. Referring to it as *Xochimilco*—after our explicit definition—ensures clarity without redundancy.

We hope this explanation addresses the concern, but we are happy to make further adjustments if needed.

**Line 144.** Indicate in the Cantera Oriente that it is a lake, lagoon, pool, etc. etc

Thank you for your suggestion. We have clarified that La Cantera Oriente (LCO) is an artificial pond.

**Line 155.** Similar to those of the old Lake Xochimilco…….

Thank you for this suggestion. In this case, we agree that using *Lake Xochimilco* is more appropriate because we are referring to the historical lake rather than our specific study site. We have updated the manuscript accordingly.

**Line 174.** Ages of the axolotls

Thank you for your observation. We have clarified in the manuscript that detailed information about the axolotls, including their age, mass, and length, is provided in Table S2.

Line 213. Age of the axolotls?
Line 224. Age of the axolotls?

Thank you for your comment. We have now included the age ranges for both male and female axolotls in each of our study areas to provide additional clarity.

Line 226-230. This information is not relevant

Thank you for pointing this out. While we believe that highlighting the involvement of local stakeholders and their cultural practices in the conservation effort is important, we have removed this section to maintain a stronger focus on the scientific content of the manuscript.

**Line 244.** Indicate which aquatic system is in (artificial wetland) LCO and in (lake) Xochimilco.

Thank you for your comment. The characteristics of each wetland system (artificial wetland for LCO and lake for Xochimilco) are already specified in the "Study Areas" section of the manuscript (Lines 158 – 159), where we describe the study locations. To avoid being repetitive, we have not restated this information in this particular line or others.

**Line 167-169.** Does the age of the axolotls have an influence? Why didn't they take into account the age data, wouldn't they be important in their study, or why did they choose the age of the axolotls? Does the age influence the displacement?

Thank you for your comment. In the "Data Analyses" section (Lines 295–297), we did not specifically mention "age" by name because it is grouped under the broader category of "biological characteristics," which also includes sex, mass, and length. However, we explicitly mention age, along with other biological characteristics, in the subsequent sentence (Lines 297-299): *“Independent t-tests were used for normally distributed data (****mass****), while Mann-Whitney U tests were applied to non-normally distributed data (****age, length****, and home range estimates).”*

Please note that age was indeed an important factor in our study and was explicitly included as an explanatory variable in our movement ecology models. As detailed in the results section (Lines 427–429), we found that age significantly influenced daily travel distances in Xochimilco, with younger axolotls traveling greater distances than older ones.

**Line 300-302.** Up to this point the variable “age” is touched upon

Thank you for your comment. The variable "age" was introduced earlier in the manuscript, as explained in our previous response. Specifically, it is mentioned in the "Data Analyses" section as part of our comparisons of biological characteristics. Additionally, it was included earlier in the manuscript (Lines 302–306) within the broader category of "biological factors," which also encompasses sex, mass, and length.

To avoid any confusion, we have now edited these lines to explicitly list the variables analyzed. The updated text reads:

*“In addition to these comparisons, we used separate generalized linear mixed models (GLMMs) for each study area to examine the influence of biological (age, sex, mass, and length), temporal (time of day and days since release), and environmental (temperature) factors on axolotl movement, specifically daily and hourly travel distances.”*

We hope this revision clarifies the inclusion and importance of age as a variable in our analyses.

**Line 323.** How many days after the release was this organism recaptured on May 24, what year?

Thank you for your comment. To provide clarity, we have now included the specific recapture years for all axolotls mentioned in the study, including the organism recaptured on May 24.

**Line 326-328.** These comments should be expressed in the discussion

Thank you for this observation. We believe that the mortality of our study animals is an important finding and should be reported in the results section. These events provide direct evidence of predation, which is an important aspect of our findings. However, we have further elaborated on the implications of this mortality in the discussion (Lines 578–584).

**Line 334.** Indicate Artificial wetland

Thank you for your comment. By this point in the manuscript, we have clearly established in the "Study Areas" section that LCO is an artificial wetland. To maintain readability and avoid redundancy, we have chosen not to repeat this information every time LCO is mentioned in the results and discussion sections. Instead, we rely on the descriptions provided earlier in the manuscript to ensure readers are adequately informed about the characteristics of the study areas. We believe this approach allows the text to remain concise while ensuring readers are adequately informed.

**Line 415-417.** You mention predation by herons on your organisms, however you do not discuss that this area of Mexico City is a place where birds migrate mainly in the winter, which would be a limitation for the presence of birds.

Thank you for this excellent observation. We appreciate your insight regarding bird migration patterns in this area of Mexico City, particularly the increased presence of birds during the winter. Based on your comment, we have updated the discussion to highlight that great egrets (*Ardea alba*) are migratory birds, with their abundance peaking in central Mexico during winter. However, we also note that these birds may be seen year-round in both study areas, including LCO, based on authors' observations. This revision provides additional context to predation risks and acknowledges the seasonal dynamics of bird populations.

**Line 418-427.** Although your study and that of Ambystoma maculatum do not mention a relationship with the presence of Lemna gibba, you have to look for that relationship with this factor, it could be the low amount of dissolved oxygen in the water, the pH, etc. etc. physicochemical parameters that can be altered by the presence of plant cover.

Thank you for your insightful comment. We agree that the presence of *Lemna gibba* could potentially influence key physicochemical parameters such as dissolved oxygen levels and pH, which may, in turn, impact axolotl distribution. While our study did not measure these parameters, we have now revised the discussion to acknowledge this possibility and highlight the need for future research to investigate the causal relationship between duckweed cover and microhabitat suitability for axolotls.

**Line 431-434.** Discuss that these areas, in addition to being a refuge from predation, are a prey niche for axolotls, since they function as reservoirs of phytoplankton and zooplankton, attracting various groups of vertebrates and invertebrates that function as prey for axolotls.

Thank you for this insightful suggestion. We agree that aquatic vegetation not only serves as a refuge from predation but may also function as a prey niche for axolotls. In response to your comment, we have revised the discussion to highlight how aquatic vegetation, such as *Myriophyllum aquaticum* and *Eichhornia crassipes*, can act as reservoirs of phytoplankton and zooplankton, which attract various groups of vertebrates and invertebrates that are potential prey for axolotls (Lines 461 – 464).

**Line 556-562.** And what can you say about the pollutants in the water? It is known that the Xochimilco Lake area is an agricultural zone, and they use agrochemicals that are dumped into the canals by leaching, affecting the survival of amphibians that have permeable skin.

Thank you for your comment. We agree that pollutants, particularly agrochemicals from agricultural activities in Lake Xochimilco, are an important factor that can affect amphibian survival due to their permeable skin. However, we believe that discussing pollution in this section, which focuses on animal behavior and predation risks, may not be ideal.

To address this point, we have included a discussion about the impact of water pollution in a more appropriate section (Lines 629–632), where environmental variables such as water quality are already mentioned. The revised text reads:

*"Most of Lake Xochimilco faces risks of agrochemical pollution due to agricultural activities, but restored chinampas that avoid agrochemical use provide critical refuges and demonstrate sustainable conservation potential. Continued monitoring and comparison of environmental variables such as temperature and water quality will be crucial in refining the use of LCO as a complementary conservation tool alongside Xochimilco."*

*In the abstract authors should mention some quantitative data, not only trends.*

Thank you for your valuable feedback. In response to your comment, we have revised the abstract to include specific quantitative data. The updated abstract now includes key findings, such as the mean home ranges for axolotls in both study areas (2,747 m² in La Cantera Oriente and 382 m² in Xochimilco), temperature ranges where movement peaked (16–17°C in Xochimilco and 15.5–16.5°C in La Cantera Oriente), and daily distances traveled by females and males in La Cantera Oriente (86.75 m/day and 54.33 m/day, respectively).

***Line 99-101.*** *What about the disadvantages? Limited range, weather conditions, organisms behavior etc. elaborate on the disadvantages and advantages of implementing this tool.*

Thank you for your comment. We have expanded the text to acknowledge the limitations of VHF telemetry, including restricted signal range, limited battery life, potential malfunctions, susceptibility to adverse weather conditions, and high costs. Additionally, we now highlight the mortality risk associated with transmitter implantation in small species like salamanders and note that this risk may be mitigated by extending post-surgery recovery time and using smaller transmitters. However, we emphasize that no mortality was observed in our study or in previous studies involving axolotls with VHF transmitters. To further clarify this, we have revised the Ethics Statement to explicitly state that all axolotls survived the implantation procedure and monitoring period.

***L415-417*** *Information has been previously reported in the ms.*

Thank you for your comment. We acknowledge that this information has been previously introduced in the manuscript. To improve clarity and avoid redundancy, we have revised the opening of the Discussion as follows:

*Our study provides insights into the movement behaviors and home range sizes of captive-bred axolotls released in an artificial wetland (LCO) and a restored chinampa within their native habitat, Lake Xochimilco.*
